# Neuromuscular transmitter candidates of a centipede (*Lithobius forficatus*, Chilopoda)

**DOI:** 10.1186/s12983-018-0274-9

**Published:** 2018-08-01

**Authors:** Hendrik Langeloh, Hannah Wasser, Nicole Richter, Gerd Bicker, Michael Stern

**Affiliations:** University of Veterinary Medicine Hannover, Division of Cell Biology, Bischofsholer Damm 15/102, D-30173 Hannover, Germany

**Keywords:** Acetylcholine, Glutamate, GABA, Neuromuscular junction, Synapse

## Abstract

**Background:**

The neuromuscular junction is the chemical synapse where motor neurons communicate with skeletal muscle fibers. Whereas vertebrates and many invertebrates use acetylcholine as transmitter at the neuromuscular junction, in those arthropods examined up to now, glutamate and GABA are used instead. With respect to taxon sampling in a phylogenetic context, there is, however, only a limited amount of data available, focusing mainly on crustaceans and hexapods, and neglecting other, arthropod groups. Here we investigate the neurotransmitter equipment of neuromuscular synapses of a myriapod, *Lithobius forficatus*, using immunofluorescence and histochemical staining methods.

**Results:**

Glutamate and GABA could be found colocalised with synapsin in synaptic boutons of body wall and leg muscles of *Lithobius forficatus*. Acetylcholinesterase activity as a marker for cholinergic synapses was found abundantly in the central nervous system and also in some peripheral nerves, but not at neuromuscular junctions. Furthermore, a large number of leg sensory neurons displayed GABA-immunofluorescence and was also labeled with an antiserum against the GABA-synthesizing enzyme, glutamate decarboxylase.

**Conclusions:**

Our data indicate that glutamate and GABA are neurotransmitters at *Lithobius forficatus* neuromuscular junctions, whereas acetylcholine is very unlikely to play a role here. This is in line with the concept of glutamate as excitatory and GABA as the main inhibitory neuromuscular transmitters in euarthropods. Furthermore, we have, to our knowledge for the first time, localized GABA in euarthropod leg sensory neurons, indicating the possibility that neurotransmitter panel in arthropod sensory systems may be far more extensive than hitherto assumed.

## Background

The evolution of animals has involved marked changes in morphology and the appearance of new features. Since cellular communication is important to organismic functioning, a phylogenetic analysis of the cellular distribution of chemical messenger molecules may be a logical way to gain additional insights into the evolutionary history.

For example, electrophysiological experiments and immunocytochemical labeling of crustacean and hexapod nervous systems (e.g. [1–6]) have demonstrated that glutamate is the most likely fast neurotransmitter at excitatory synapses onto skeletal muscles. This is in contrast to the vertebrates and many other bilaterian invertebrates which use acetylcholine instead (e.g. [6-11]). See Table 2 on page 92 in [11] for review). Thus, the presence of glutamate as the exclusive fast excitatory skeletal neurotransmitter may be a synapomorphy of the Euarthropoda. So far, this hypothesis has only been addressed in a very limited number of neurophysiological and genetic model organisms such as lobster [1], locust [2, 4], and fruit fly [3, 5], but not in other arthropod taxa. Our finding, that the onychophorans which are the most likely sister group [12], but not direct members of the euarthropod taxon, signal with both acetylcholine and glutamate to the body wall muscles, provides additional evidence that the transmitter phenotype of the excitatory neuromuscular junctions can be used as phylogenetic character of Arthropoda [13].

Some muscle fibers in arthropods are not only innervated by excitatory synapses, in which glutamate is the neurotransmitter, but also by inhibitory motor neurons, which use gamma-aminobutyric acid (GABA) [14–17]. During the evolution of arthropod locomotion, GABAergic inhibitory neuromuscular transmission may have been an advantageous character (review [18]), but was also lost in several taxa. In Hexapoda, it appears to be present in some basal groups such as Zygentoma, Odonata, or Orthoptera, but is missing in several holometabolous insects [19]. On the input side of the arthropod nervous system and again, in contrast to the vertebrates, acetylcholine (ACh) seems to be the predominant classical transmitter in mechanosensory and chemosensory neurons of crustaceans [20–23] and hexapods [24–29].

In spite of a wealth of neuroanatomical investigations about the chemical architecture of the arthropod central nervous system [30], at present the taxon sampling for neurotransmitter candidates in the peripheral nervous system is rather sparse. In particular, the terrestrial arthropod taxon of Myriapoda represents a rather understudied taxon [31, 32].

**Fig. 1:**
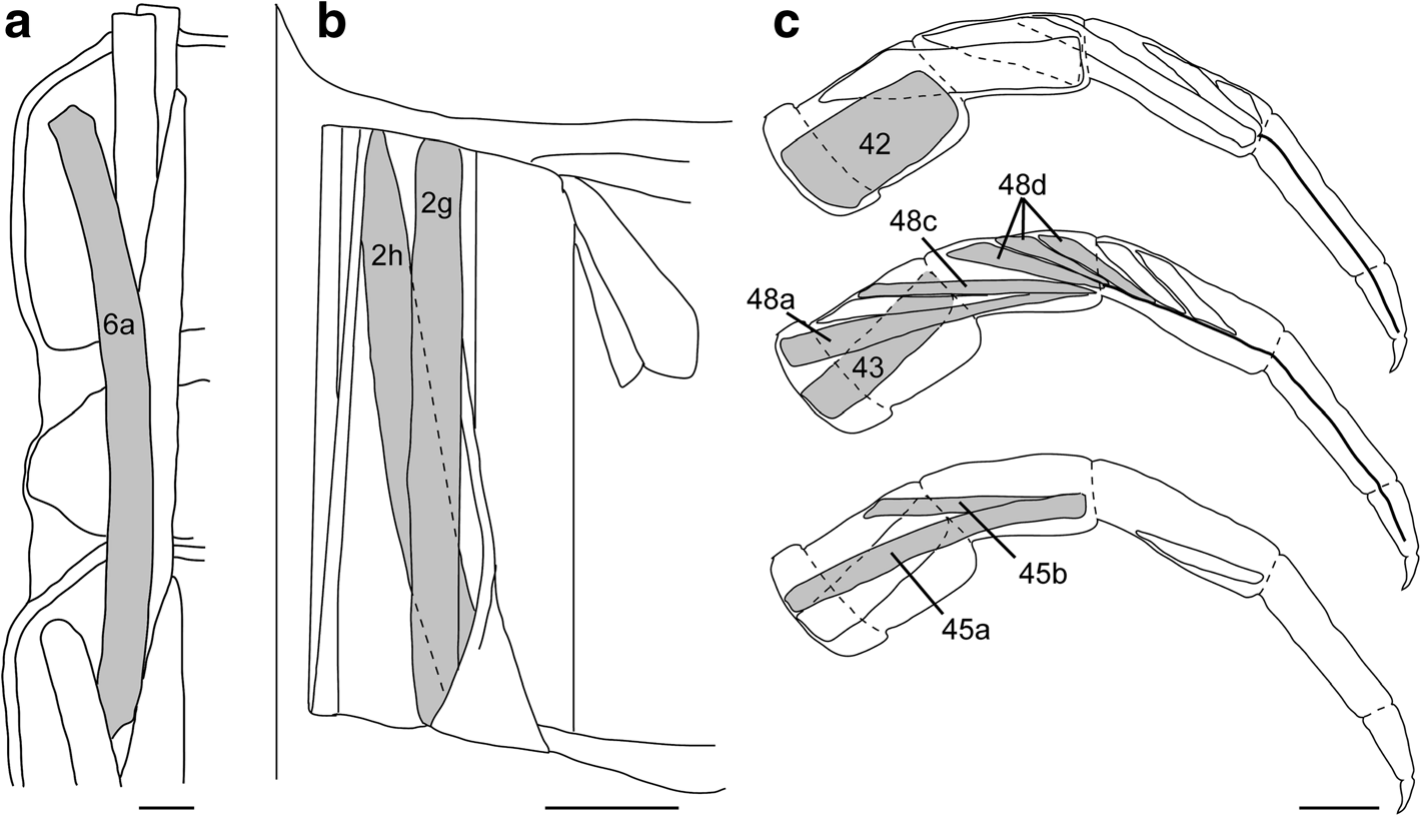
Schematic diagrams of the muscles, where neuromuscular junctions were analyzed (adapted and simplified from Rilling, 1960). **a** Right half of 2 ½ tergites with dorsal body wall muscles. **b** left half of a single sternite with ventral body wall muscles. **c** posterior ts view of a right walking leg in three different planes (anterior, medial, posterior) with leg flexor muscles. Muscles analyzed in this study are shaded in grey and numbers according to Rilling (1960) are indicated. **a**, **b**: anterior is to the top, **c** dorsal is to the top. All scale bars 500 μm

We are currently collecting comparative cytochemical data of non-model arthropods, and focus here on the peripheral nervous system of the chilopod *Lithobius forficatus*. This animal figured prominently in von Holst’s [33, 34] leg amputation studies on arthropod locomotion that contributed to the drafting of his principle of central coordination. The internal anatomy has been described by Rilling [35]. Within the 15 leg bearing segments, the muscles and their innervation are arranged in invariant patterns facilitating neuroanatomical investigations in whole mounts. Neuromuscular innervation of body wall and appendages is supplied by eight pairs of peripheral nerves originating from each of the segmental ganglia of the ventral nerve cord. Ventral body wall musculature (Fig. 1b) is innervated by nerves 1 and 7, dorsal body wall muscles (Fig. 1a) by nerves 3 and 6–8, and muscles of the walking legs (Fig. 1c) are innervated by the largest nerve (4), and the much smaller nerve 5. In more modern times, a study of the ventral nerve cord of *Lithobius forficatus* revealed large numbers of GABA-immunoreactive processes in the connectives but also in nerve roots [19]. Quite recently, a study of serotonergic neurons in the ventral nerve cord ganglia of the same species and other centipedes [36] has contributed to the understanding of phylogenetic relationships within the arthropods.

In the present study, we describe glutamate-IR synaptic terminals on the muscles of the leg and body wall, suggesting glutamate as excitatory neuromuscular transmitter. Similar to crustaceans, hexapods and arachnids, we find also GABA immunoreactive synaptic boutons on most skeletal muscles. Using antisera against GABA and its biosynthetic enzyme glutamic acid decarboxylase (GAD), we additionally identify also immunoreactivity in certain subcuticular sensory neurons. To the best of our knowledge, this is the first evidence in an arthropod species for GABA serving not only as neurotransmitter in the CNS or neuromuscular system, but also in certain sensory neurons.

## Methods

### Animals

All chemicals were purchased from Sigma (Merck, Darmstadt, Germany), if not stated otherwise. A total of 63 specimens of *Lithobius forficatus* (Linnaeus, 1758) were collected locally under loose bark or rocks in the Eilenriede forest of Hannover, Germany, and kept in 135 mm Petri dishes at 4 °C until dissection. Even when collected in winter at temperatures below 0 °C, centipedes were moving as fast as at room temperature. Animals were decapitated and dissected in cold phosphate buffered saline (PBS: 10 mM sodium phosphate, 150 mM NaCl, pH 7.4) or PBS with the addition of 100 mM sucrose and 5 mM EDTA. The latter helped to improve tissue integrity and intensity of glutamate or GABA immunolabeling at synapses. To expose neuromuscular synapses, animals were cut in portions consisting of 3–4 segments, cut open laterally, and pinned out internal side up in a Sylgard-lined Petri dish. Guts and parts of the tracheal system and fat body were removed before fixation. Legs were separated and cut approximately into anterior and posterior halves with iridectomy scissors to allow for access of chemicals and antibodies and allowing frontal/rear view into the leg. A schematic drawing of investigated muscle fibres in the body wall and legs, numbered according to Rilling [35] is shown in Fig. 1.

In some cases, ventral nerve cord ganglia were dissected out after fixation, embedded into 7% low melting agarose (Roth, Karlsruhe, Germany) and sectioned (horizontal or sagittal plane) at 50 μm on a vibrating microtome (Leica VT 1000S). Each labeling method was repeated at least three times on independent specimens.

### Histochemistry of acetylcholinesterase

Tissue was fixed in 4% paraformaldehyde in PBS for 30 min at 4 °C. After three rinses in PBS, the cuticle was partially removed. Tissue was permeabilised in 0.3% saponin in PBS for 1 h at room temperature and processed for acetylcholinesterase staining (AChE) using a modification of the method of Karnovsky and Roots [37] with 3 mg acetyl-thiocholine/ml Tris/maleate buffer, pH 5.85, at room temperature until dark brown staining was visible in ventral nerve cord ganglia (usually ~ 30 min). As a specificity control, a few specimens were pre-incubated for 30 min in 30 μM eserine in Tris/maleate buffer, followed by incubation in staining solution with 30 μM eserine alongside preparations processed without eserine. In eserine-treated preparations, staining was completely absent.

### Immunofluorescence

Tissue was dissected as described above and fixed for one hour at room temperature in 4% paraformaldehyde (for GAD immunolabeling) or for two hours on ice in 4% paraformaldehyde plus 0.05% glutaraldehyde in PBS (for neuromuscular junctions) or 4% PFA plus 0.2% glutaraldehyde (for CNS). Preparations were either processed as whole mounts or as vibratome sections. In any case, tissue was permeabilised in 0.3% saponin in PBS for 1 h at room temperature after fixation or sectioning, followed by three washes in PBS with 0.1% Triton X-100 (PBS-T). After blocking in 5% normal goat serum in PBS-T, specimens were incubated in the primary antiserum overnight at 4 °C. The antibody against synapsin (SYNORF1, 3C11, supernatant [38]), obtained from the Developmental Studies Hybridoma Bank, Iowa, was diluted 1:10–1:20 in blocking solution. Polyclonal antisera against glutamate (Sigma cat No. G6642) or GABA (Sigma cat.No. A2052) were added at 1:2000. A polyclonal antiserum against glutamate decarboxylase (GAD, Sigma cat No. G5163) was used at 1:1000. This antibody required a denaturation step by dehydrating and rehydrating the tissue through an ethanol series (50, 70, 85, 96% for 10 min each) before blocking. After several washes in at least two hours, a secondary antibody cocktail (goat anti-mouse-AlexaFluor488, Thermo Fisher Scientific, for synapsin, and goat anti-rabbit-AlexaFluor568 for anti-glutamate, anti-GABA, or anti-GAD) was applied 1:250 in blocking solution with 0.1 μg/ml DAPI (4′,6-Diamidino-2-Phenylindole) as a nuclear marker at 4 °C overnight. In a few preparations labelled for GAD-IR, we used a donkey anti-rabbit-AlexaFluor488 secondary antibody instead.

For co-labeling of acetylcholinesterase and synapsin-immunoreactivity (synapsin-IR), AChE-stained preparations were blocked, and incubated in SYNORF1 antibody as above, and visualized by goat anti-mouse-alexaFluor568, 1:250, as above. After washing, specimens were cleared in glycerol (50% in PBS, 90% in PBS) and mounted in 90% glycerol containing 4% sodium propyl gallate as an anti-fading agent. Preparations were viewed with a Zeiss Axioscope. Images were acquired with an Axiocam 506 color and image acquisition system (Zeiss Axiovision). Confocal images were acquired using a Leica TCS SP5 confocal laser-scanning microscope using Leica LCS software. Images were subsequently merged with ImageJ (W.S. Rasband, U.S. National Institutes of Health, Bethesda, MD, http://rsb.info.nih.gov/ij/) for maximum intensity projections of z-stacks. Anterior is to the left in all figures, if not explicitly stated otherwise.

### Antibody specificity

BSA (bovine serum albumin)-glutaraldehyde conjugates of glutamate and GABA were prepared according to [39]. Working dilutions of the antiserum were mixed 1:1 with each conjugate or a non-conjugated BSA-solution and incubated for four hours at room temperature before applying them to the tissue instead of the normal antibody solution. After development with fluorescent secondary antibodies, no red immunofluorescence was detected, whereas green synapsin-IR was not impaired. Antibody cross-reactivity was determined by dot blot immunoassay as described in [5], using the conjugates of glutamate and GABA, respectively, in a 1:10 dilution series. The GABA antiserum was over 1000-fold more sensitive to GABA than to glutamate, and the glutamate antibody was over 100 fold more sensitive to glutamate than to GABA.

The antibody 3C11 (SYNORF1) has been generated and extensively characterized by Klagges et al. [38]. Mice were immunized with fusion protein of Glutathion-S-Transferase and recombinant synapsin, cloned from cDNA of *Drosophila* heads. The antibody recognizes both isoforms of the presynaptic protein synapsin in *Drosophila* and many other invertebrate species including Chilopoda [40]. In Western Blots performed on *Lithobius forficatus* homogenates (data not shown) it labels a strong band at ~ 60 kDa, a band just below 150 kDa, and two much weaker bands in between. This corresponds to the bands labeled in the original publication on *Drosophila* synapsins [38]. The antibody against GAD (Sigma, catalogue number G5163), was raised against the highly conserved C-terminal of both the 65 kDa and 67 kDa isoforms of vertebrate GAD. In our Western Blot analysis (data not shown) it recognized a strong band close to 60 kDa and another band between 100 and 150 kDa, which is close to the values reported for other invertebrates, e.G. *locusta*, [41]: ~ 50 and ~ 100 kDa. The higher molecular weight signal most likely represents protein dimers since both GAD isoforms are reported to form homodimers in vivo and in vitro [42]. In our preparations of ventral nerve cord ganglia and legs it labels the same structures as the GABA antiserum (see Results).

## Results

### Ventral nerve cord ganglia

**Fig. 2:**
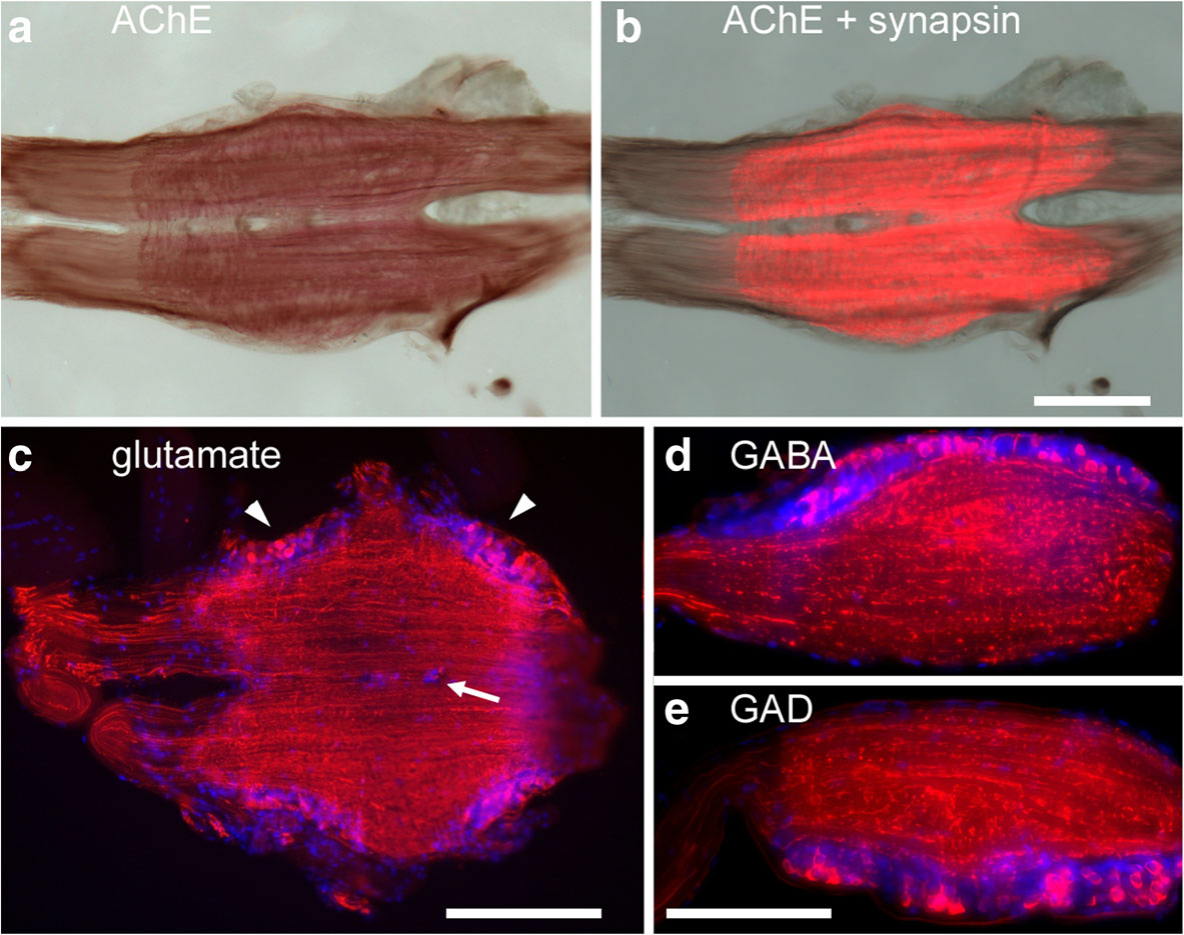
Horizontal vibratome sections of ventral nerve cord ganglia. **a** Acetylcholine esterase staining (*brown*). **b** Merge of acetylcholine esterase staining (*brown*, same section as in **a**) and synapsin-IR (*red*). **c** Glutamate-IR (*red*) and nuclear labeling (DAPI, *blue*). *Arrowheads* point to clusters of glutamate-IR cell bodies, *arrow* points to midline cell bodies. **d** Left hemiganglion labeled with GABA antibody (*red*) and DAPI (*blue*). **e** right hemi ganglion labeled with glutamate decarboxylase antibody (GAD, *red*) and DAPI (*blue*). Anterior is to the left in all panels. Scale bars 200 μm, scale bar in **b** also applies for **a**, scale bar in **e** also applies for **d**

Like in other arthropods, segmental ventral nerve cord ganglia of *Lithobius forficatus* consist of a cell body rind surrounding the core neuropil (Fig. 2). On sections of ganglia, the neuropil is stained strongly for acetylcholinesterase (Fig. 2a, b). Also, many fibers projecting into the connectives are stained outside the neuropil, the delimitations of which can be clearly detected by synapsin –IR (Fig. 2b). The cell body rind was not stained for AChE or synapsin-IR. The ganglia are fusions of two bilaterally symmetric hemiganglia, which is evident from the partial lack of acetylcholinesterase and synapsin-IR along the midline, and the presence of DAPI-labeled nuclei near the midline (Fig. 2c). Glutamate- and GABA-IR were both abundant in ventral nerve cord ganglia (Fig. 2c, d). In addition to the dense immunolabeled meshwork within the neuropil, some axons were labeled in the connectives. Several glutamate-IR cell bodies appeared loosely clustered (Fig. 2c). Cell bodies labeled for GABA (Fig. 2d) or the GABA synthetizing enzyme, glutamate decarboxylase (GAD, Fig. 2e) appeared to be more uniformly distributed within the cell body rind (Fig. 2d).

### Neuromuscular junctions

**Fig. 3:**
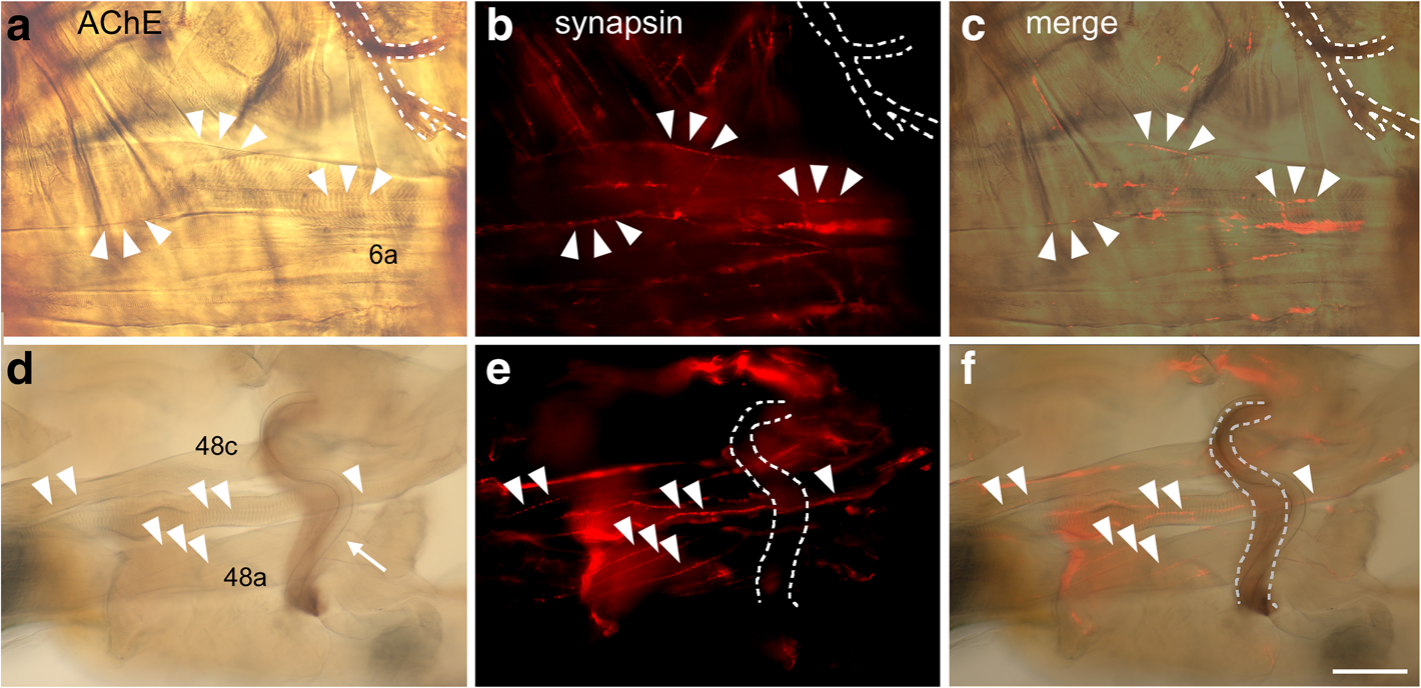
Localisation of acelylcholine esterase staining (*brown*) and synapsin-IR(*red*). **a**-**c** Dorsal body wall muscle 6a and peripheral nerve stained for acetylcholine esterase (**a**) and immunolabeled for synapsin (**b**), **c** is a merge of **a** and **b**. Peripheral nerve is outlined in white, selected synaptic boutons from B are marked with *arrowheads* in all panels. **d**-**f**: Tibia of a walking leg stained for acetylcholine esterase (**d**) and immunolabeled for synapsin (**b**), **f** is a merge of **d** and **e**. Peripheral nerve 4 is outlined in white, selected synaptic boutons from **e** are marked with *arrowheads* in all panels. *Arrow* points to a trachea accompanying the leg nerve. Note absence of colocalization of AChE and synapsin. Muscle numbers are indicated. Anterior is to the left in **a**-**c**, distal is to the left in **d**-**f**. Scale bar 100 μm

**Fig. 4:**
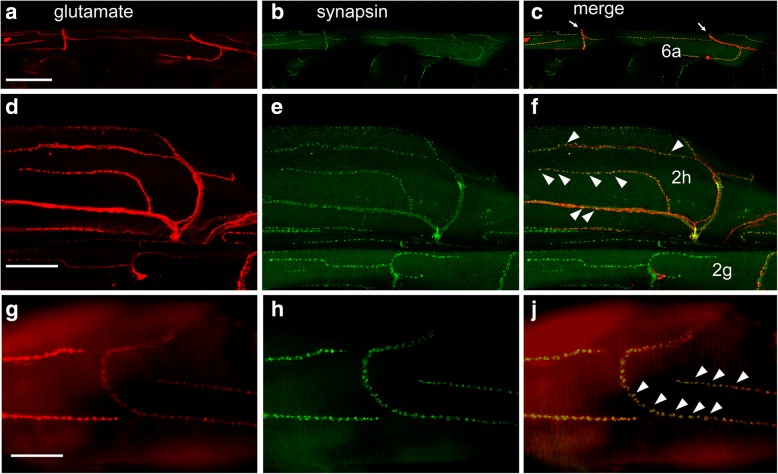
Colocalisation of synapsin- with glutamate-IR at body wall neuromuscular junctions. **a**-**c**: Confocal images (maximum projection of 18 images at 1 μm intervals) of dorsal body wall muscles 6a (intersegmental muscles of a segment with long tergite) labeled with antibodies against glutamate (**a**) and synapsin (**b**) displaying numerous co-labeled boutons (**c**) *Arrows* point to two motor nerves innervating the muscle. **d**-**f**: Confocal images (maximum projection of 28 images at 1 μm intervals) of ventral body wall muscles 2 h and 2 g labeled with antibodies against glutamate (**d**) and synapsin (**e**) displaying numerous co-labeled boutons (**f**, *arrowheads*).). **g**-**j**: Confocal images (maximum projection of 11 images at 1.6 μm intervals) of a ventral body wall muscle labeled with antibodies against glutamate (**g**) and synapsin (**h**). Presynaptic sites appear as aggregates of several small dots, most of which are co-labeled by both antibodies (**j**, *arrowheads*). Anterior is to the left in all panels. Scale bar: **a**-**c** 100 μm, **d**-**f** 50 μm, **g**-**j** 20 μm

**Fig. 5:**
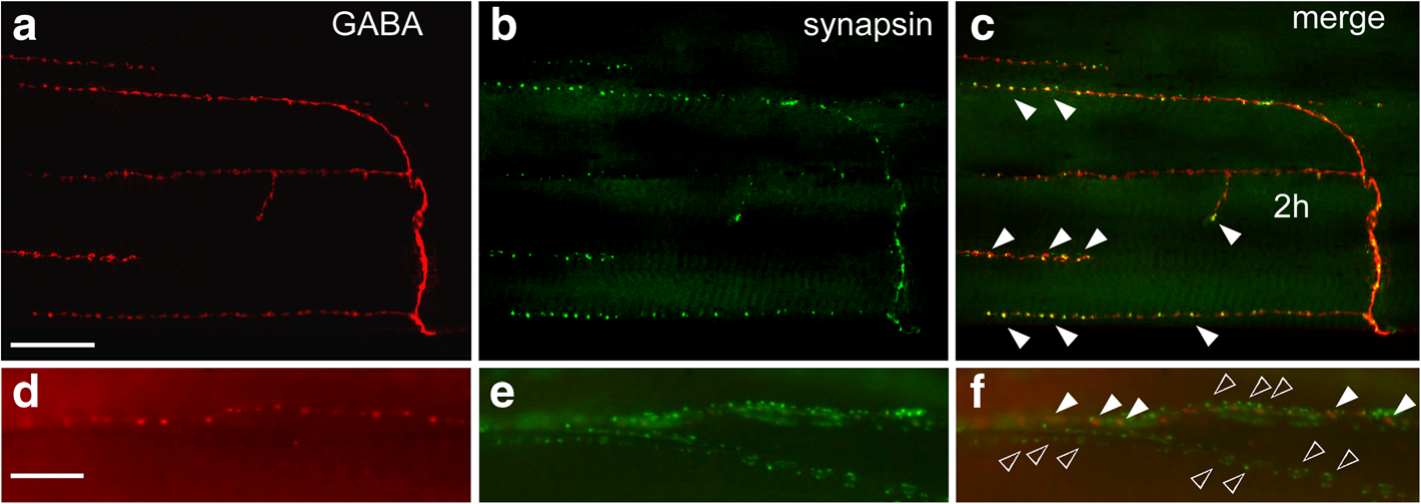
Colocalisation of synapsin- with GABA-IR at body wall neuromuscular junctions. **a**-**c**: Confocal images (maximum projection of 18 images at 1 μm intervals) of ventral body wall muscle 2 h labeled with antibodies against GABA (**a**) and synapsin (**b**) displaying numerous co-labeled boutons (**c**). **d**-**f**: Fluorescence images of lateral body wall muscle 34 (a dorsoventral muscle not depicted on Fig. 1). Presynaptic sites appear as aggregates of several small synapsin-IR dots, some of which are co-labeled against GABA (**f**, *white arrowheads*), whereas most of them are not labeled for GABA (*hollow arrowheads*). Anterior is to the left in **a**-**c**, dorsal is to the left in **d**-**f**. Scale bar: **a**-**c** 100 μm, **d**-**f** 10 μm

**Fig. 6:**
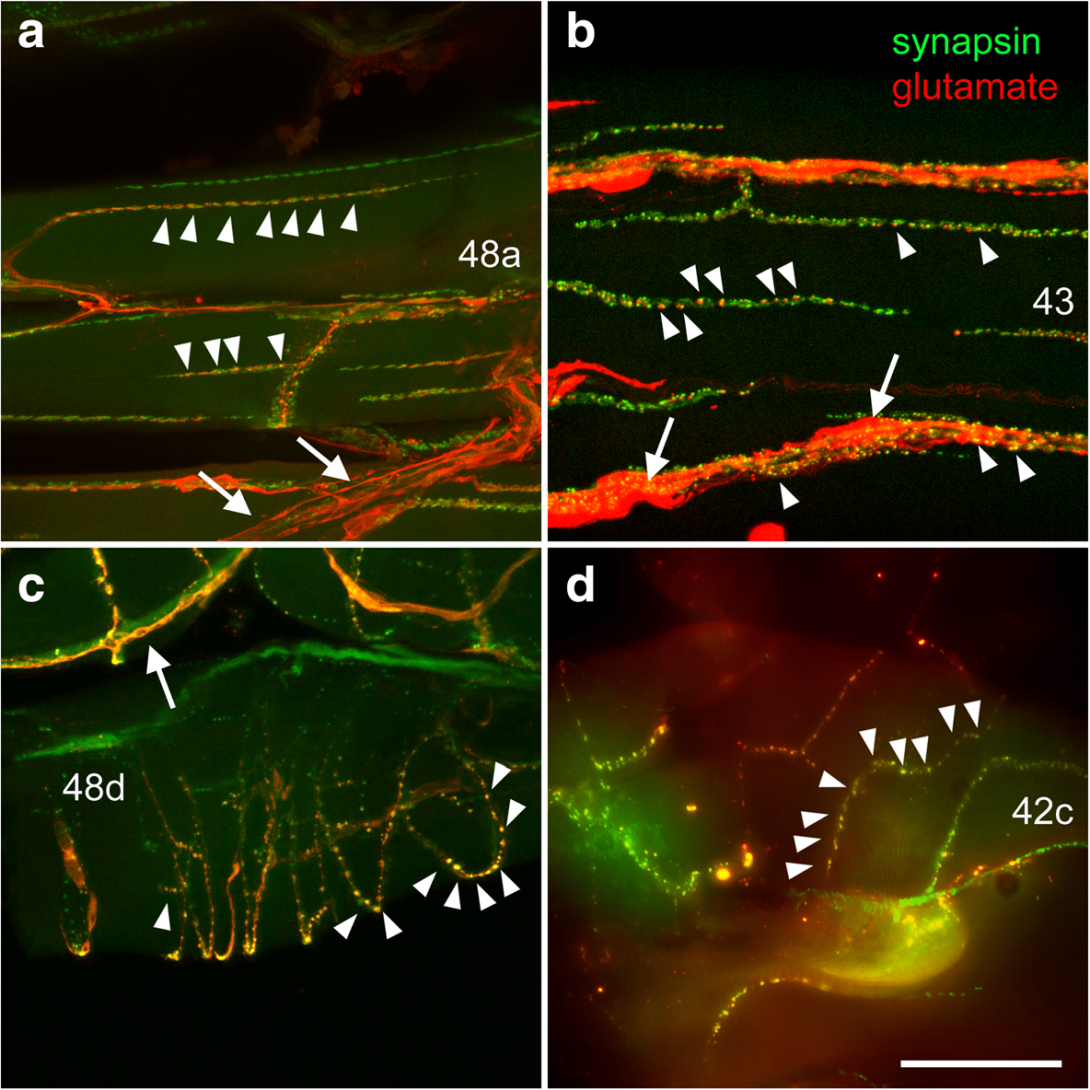
Colocalisation of synapsin- (green) with glutamate-IR (red) at leg neuromuscular junctions. **a** Merged confocal images (maximum projection of 33 images at 1 μm intervals) of muscle 48a in the femur, displaying numerous co-labeled boutons (*arrowheads*). A motor nerve containing several fibers labeled for glutamate is indicated by *arrows*. **b** Merged confocal images (maximum projection of 25 images at 1 μm intervals) of muscle 43 in the trochanter 2, displaying numerous co-labeled boutons (*arrowheads*) and strong glutamate-immunoreactivity surrounding synapsin-IR motor endings, indicating presumptive glia cells (*arrows*). **c** Merged confocal images (maximum projection of 28 images at 1 μm intervals) of muscle 48d (claw flexor) in the femur displaying numerous co-labeled boutons (*arrowheads*). Presumptive glial cells surrounding synapsin-IR motor endings are strongly labeled for glutamate (*arrow*). **d** Merged fluorescent images of muscle 42c in the trochanter 2 displaying numerous co-labeled boutons (*arrowheads*). Numbers refer to muscle numbers. Distal is to the right in all panels. Scale bars: **a** 100 μm, **b**-**d** 50 μm

**Fig. 7:**
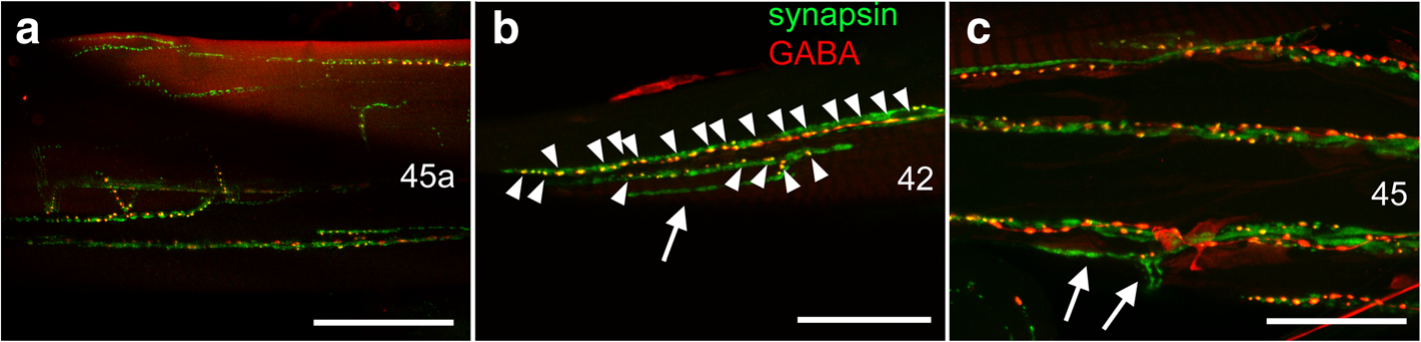
Colocalisation of synapsin- (green) with GABA-IR (red) at leg neuromuscular junctions. **a** Merged confocal images (maximum projections of 35 images at 1 μm intervals) of muscle 45a. **b** Merged confocal images (maximum projections of 35 images at 1 μm intervals) of muscle 42 displaying numerous co-labeled boutons (*arrowheads*) and junctions only labeled against synapsin (*arrows*). **c** Merged confocal images (maximum projection of 30 images at 1 μm intervals) of muscle 45 displaying numerous co-labeled boutons and junctions only labeled against synapsin (*arrows*). Numbers refer to muscle numbers. Distal is to the right in all panels. Scale bars: **a** 100 μm, **b**, **c** 50 μm

Anatomically, the neuromuscular system of *Lithobius forficatus* has been extensively described by Rilling [35] and we follow his nomenclature for muscles here (Fig. 1). Using anti-synapsin immunolabeling, neuromuscular junctions could be identified on all skeletal muscles (examples in Figs. 3, 4, 5, 6, 7). Neuromuscular synaptic sites appeared as chains of varicosities (boutons) along nerve branches spaced ca. 3–8 μm apart. Each muscle fiber was innervated by one to three nerve branches, which projected parallel to the longitudinal extension of the muscle. Colocalisation of synapsin-IR and neurotransmitter-IR or acetylcholinesterase staining was employed to identify neuromuscular transmitter candidates.

### Neuromuscular junctions on body wall muscles

Most peripheral nerves were stained to a lesser extent by AChE (Fig. 3a, d). On muscles, however, no specific staining was observed. In particular, neuromuscular junctions labeled for synapsin-IR (Fig. 3b, e) displayed no corresponding AChE-staining both in body wall (Fig. 3c) and leg muscles (Fig. 3f).

Both synapsin and glutamate-IR were found on every body wall muscle examined. The body segmentation of *Lithobius forficatus* is homonomous on the ventral side, but heteronomous on the dorsal side, with alternating large and small tergal sclerites. For this reason, some dorsal intersegmental muscles connecting large tergites are very long (Fig. 1a), and receive innervation from up to three adjacent ventral nerve cord ganglia [35, 43]. For instance, on dorsal muscle 6a, innervation appears to originate from two primary nerves (originating from adjacent ganglia) without overlap (Fig. 4a-c). Neuromuscular connections appeared as *en passant* synapses where nerve terminals extending along the muscle surface produced a row of more or less equidistant bouton-like synaptic endings. Nerve terminals were only sparsely branched to contact each fiber of the innervated muscle by a single or very few rows of synapsin-IR spots (Fig. 4). At higher magnification, it became apparent that each spot often consisted of a cluster of 2–4 closely adjacent boutons (Fig. 4h), most of which displayed colocalisation of synapsin- and glutamate-IR (Fig. 4j).

Similar observations were made for GABA-IR, which was also colocalised with synapsin-IR spots (Fig. 5). However, not every synapsin-IR spot colocalised with GABA-IR. On several muscles, there were motor nerve branches completely devoid of GABA-IR, and even within branches with colocalisations only a fraction (~ 25%) of the synapsin-IR sites were also labeled for GABA-IR (Fig. 5d-f).

### Neuromuscular junctions on walking leg muscles

In walking legs, all identified muscles were innervated by nerve terminals containing several glutamate-IR fibers (Fig. 6a) with boutons containing glutamate-IR colocalised with synapsin-IR (muscles 48a and 48d in the femur, and 42c and 43 in the prefemur are shown as examples in Fig. 6). Sometimes, strong diffuse glutamate IR was observed surrounding synapsin-IR nerve terminals (Fig. 6b, c), originating presumably from glial cells. This feature could be reduced but not completely abolished by quick dissection in ice-cold saline containing 5 μM EDTA, possibly indicating glutamate release from terminals of motor nerves damaged during the dissection process and uptake of this released amino acid by surrounding glia. Similar to the situation seen on body wall muscles, leg neuromuscular junctions appeared as chains of varicosities containing several small synapsin-IR boutons (Fig. 6c), many of which displayed colocalisation with glutamate-IR. Despite smaller size of the muscles, motor nerve terminals appeared to be more strongly branched than body wall muscles, or the number of nerve muscle contacts was enhanced by a less straight course of the nerve terminal along the muscle. For instance, the motor terminal had grown around a fiber of muscle 48d in a spiral (Fig. 6c), or resembling a spiral staircase on muscle 42c (Fig. 6d).

GABA-IR was also widely distributed in colocalisation with synapsin-IR (Fig. 7). However, in comparison to glutamate, there were fewer GABA-IR spots, and there were also numerous motor nerve branches displaying synapsin-, but not GABA-IR (Fig. 7b, c). In a few leg muscles (m44b, m45b, m47), we observed no GABA-IR on neuromuscular junctions in any preparation.

### GABA in leg sensory neurons

**Fig. 8:**
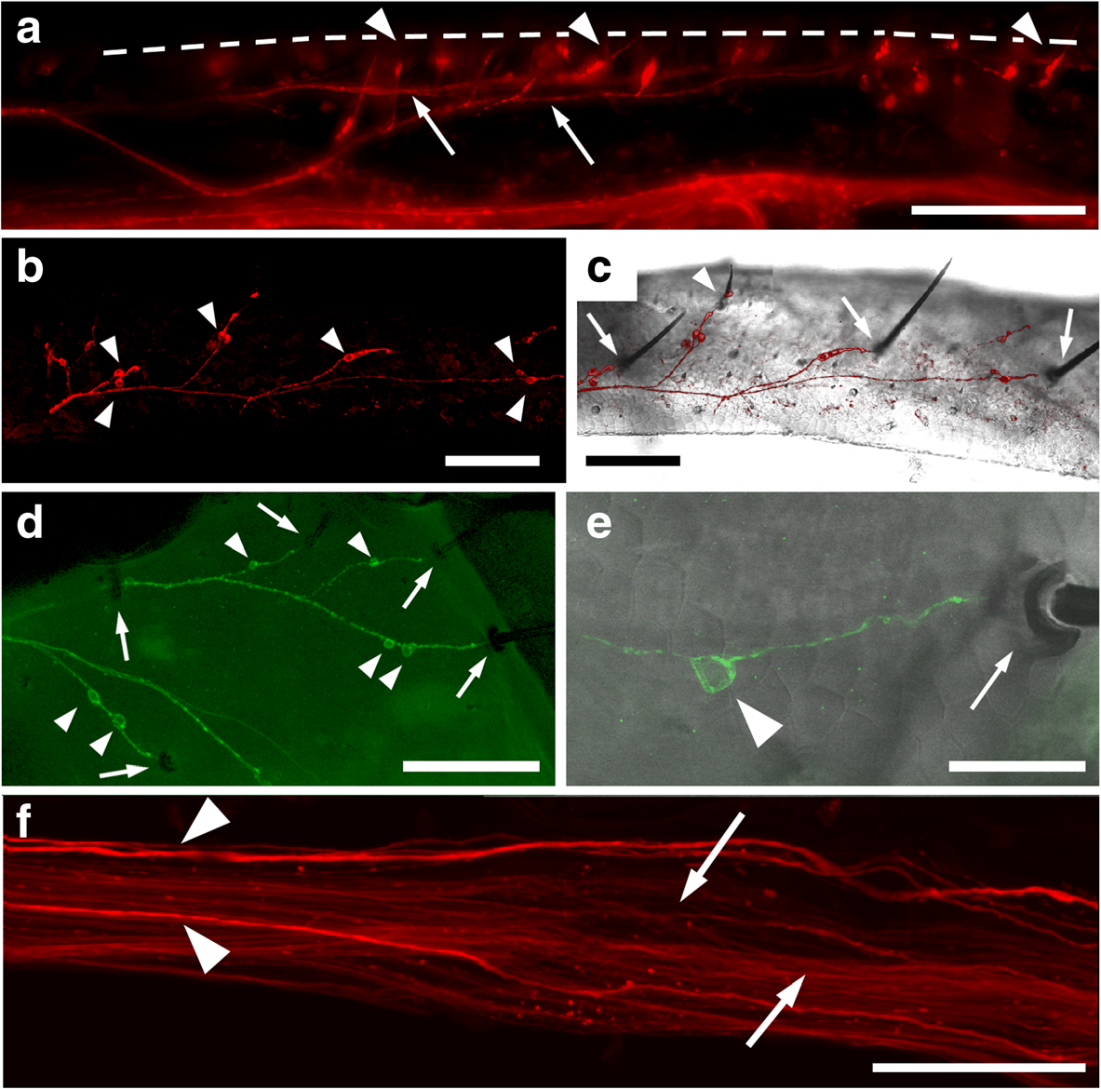
GABA and GAD in sensory neurons of walking legs. **a** Fluorescence image of GABA-IR in the tibia of a walking leg. The position of the cuticle is indicated by a *dashed line*. *Arrowheads* point to individual sensory neurons extending a dendrite through the cuticle. *Arrows* point to sensory nerve branches collecting the axons of sensory neurons. **b** Confocal image (maximum projection of 17 images at 3 μm intervals) of GAD-IRin sensory neurons (*arrowheads*) in the tibia. **c** Combination of the same confocal image as in **b** with a transillumination photomicrograph of the cuticle, displaying three large (*arrows*) and one smaller (*arrowhead*) sensilla innervated by dendrites of GAD-IR sensory neurons and corresponding sensory nerve branches (red). **d** Confocal image (maximum projection of 19 images at 1.8 μm intervals) of GAD-IR (green) in the distal part of the femur. One or two sensory neurons (*arrowheads*) extend dendrites into each of the sensilla (*arrows*) that can be seen as mdark outlines against the deliberately strong green background fluorescence. **e** Combination of a confocal image (maximum projection of 13 images at 2 μm intervals) of a GAD-IR sensory neuron (*arrowhead*) extending a dendrite into the socket of a sensillum (*arrow*). **f** Confocal image (maximum projection of 23 images at 1 μm intervals) of GABA-IR in the leg nerve (n4) in the region of the trochanter. *Arrowheads* point to large GABA-IRaxons (presumptive motor neuron axons), *arrows* point to small GABA-IR axons (presumptive afferents). Distal is to the right in all panels. Scale bars: **a** 200 μm, **b**-**d** 100 μm, **e** 20 μm, **f** 50 μm

In walking legs, GABA–IR was not confined to neuromuscular junctions and the motor nerve, but occurred also in sensory neurons of bristle or hair sensilla (Fig. 8). This was most easily visible in the tibia (Fig. 8a) because it contains fewer muscles which would obscure or prevent labeling of sensory neurons beneath, but could be found on other podomeres as well. Sensory neurons could also be labeled with an antibody against the GABA-synthesizing enzyme, GAD (Fig. 8b-e). This antibody also weakly labeled synaptic boutons on body wall and leg muscles (data not shown). One or two cell bodies sent dendrites into the socket of a sensillum (Fig. 7c-e), and sent axons along the sensory branches of the leg nerves (Fig. 8a). It appeared as is every sensory bristle was innervated by at least one GABA/GAD-IR neuron. At the level of the trochanter, numerous narrow GABA-IR fibers could be easily distinguished from three or four larger fibers which most likely represent motor axons (Fig. 8f).

## Discussion

### Neuromuscular junctions

We have shown that the CNS of *Lithobius forficatus* contains glutamate- and GABA-IR cell bodies and nerve fibers (Fig. 2). Acetylcholinesterase activity is present throughout the ganglionic neuropil, the extension of which is identified using the SYNORF1 antibody raised against *Drosophila* synapsin [38]. Neuromuscular junctions (NMJs) of body wall and leg muscles could be identified using this antibody as well. In contrast to many hexapod NMJs, which often consist of heavily branched multiterminal motor endings [5, 15, 44], where each terminal ends in a few synaptic boutons, NMJs of *Lithobius* forficatus appeared to be mostly of the *en passant* type, where a single sparsely branched motor nerve terminal extends along the muscle fiber producing numerous synaptic boutons along its extension. There appears to be a great variability in NMJ structural organization in Arthropoda [6], similar to the situation in vertebrates, where the NMJ can appear as a widely distributed structure with few long branches extending along a considerable length of muscle fiber (e.g. in frogs), or a focused multiterminal branch ending in a patch of synaptic boutons (e.g. in humans) [8]. The question, whether these structural differences might contain any phylogenetic signal awaits further investigations.

On all body wall and walking leg muscles examined, glutamate-IR could be found colocalised with synapsin-IR, indicating that glutamate is a likely neuromuscular neurotransmitter candidate. In contrast, no evidence was found for the presence of acetylcholinesterase in the vicinity of synapses, which makes any cholinergic neuromuscular transmission unlikely. The accessible nerve-muscle synapses of certain decapod crustaceans and insects helped to establish glutamate as excitatory neuromuscular transmitter in euarthropods [1–3], [rev. 6]. Since physiological evidence points towards glutamate/aspartate as excitatory neuromuscular transmitter candidates of the chelicerate *Limulus polyphemus* [45, 46] and our immunocytochemical labelings of scorpion (*Tityus stigmurus*) neuromuscular junctions show co-localization of synapsin- and glutamate-IR (unpublished), this study in a myriapod reinforces the notion, that glutamatergic excitatory transmission onto skeletal muscles may be a characteristic neurochemical feature of the euarthropods, while onychophorans use both glutamate and acetylcholine [13].

GABA-IR could be found colocalised with synapsin-IR on a large number of both body wall and walking leg muscles. This is consistent with the observation of Witten and Truman [19] who found GABA-IR fibers in all nerve roots of *Lithobius forficatus* ventral nerve cord ganglia. It also supports the view that peripheral inhibition is a rather common feature of euarthropod motor control. The existence of inhibitory motor neurons in *Lithobius forficatus* has also been suggested by Harzsch et al. [47], based on immunolocalization of GABA combined with nerve backfills in ventral nerve cord ganglia. GABAergic motor neurons are thought to speed up locomotion via selective inhibition of slowly contracting muscle fibers [6, 15], a capacity that would facilitate the rapid motility of *Lithobius forficatus*.

### GABA in sensory neurons

A surprising observation was GABA-IR in a large number of leg sensory neurons. Labeling was present not only in the cell bodies, but also in dendrites entering the sockets of sensilla and in the axons, together forming sensory branches of the leg nerve. A large number of those very small diameter GABA-IR fibers were labeled, and easily distinguished from large diameter GABAergic motor axons, in the proximal leg nerve. Our observation is supported by GABA-immunostaining in *Lithobius forficatus* ventral nerve cord ganglia by Witten and Truman [19], who report a large number of GABA-IR axons in “the nerve root just posterior to the midpoint of the ganglion”, which is the large leg nerve n4 [35]. Further support comes from our immunolabeling of the GABA-synthesizing enzyme, GAD, in leg mechanosensory neuron cell bodies (Fig. 8d-e). Alternatively, labeling could originate from GABAergic efferents contacting sensory neurons, as reported from the lyriform organ of the spider [48] or mechanosensory hair plates in the locust [49]. However, in those examples, numerous fine, varicose efferent terminals surround the sensory cells which themselves are not labeled. In our preparations, the cytoplasm (excluding the nucleus), dendrites and axons themselves are labeled. Thus, on the level of fluorescence microscopy, GABA and its synthesizing enzyme, GAD, are clearly located within the sensory cells. To our knowledge, the only other report of the possible presence of GABA in arthropod sensory cells is on the aesthetascs of freshwater prawns [50]. Outside the arthropods, GABA-IR sensory neurons can be found e.g. in the body wall annelids [51]. Acetylcholinesterase, as far as it could be detected in the vicinity of the brown pigmentation of the cuticle, could not be observed near sensory neurons. However, since output synapses of afferents are to be expected in the CNS, and not in the periphery, this does not rule out acetylcholine as a potential (co-)transmitter in these cells. There are also other exceptions from the general view that ACh serves as the main mechanosensory transmitter in the arthropod phylum. Based on immunocytochemical staining and genetic loss of function studies, histamine has been identified as a transmitter candidate in mechanosensory neurons of *Drosophila melanogaster* [52, 53]. In the spider *Cupiennius salei*, histamine may serve as a co-transmitter of ACh in mechanoreceptive organs [54].

### Conclusions

Our data, which indicate that glutamate and GABA are most likely neurotransmitters at *Lithobius forficatus* neuromuscular junctions whereas acetylcholine is very unlikely to play a role in neuromuscular transmission in this animal, have closed a gap in taxon sampling for neuromuscular transmitter identity. Similar investigations in Chelicerata [16] are still sparse and need to be extended. Nevertheless, current data are in line with the concept of glutamate and GABA being the main neuromuscular transmitters in Euarthropoda. However, more chemoneuroanatomical and neurophysiological investigations in other arthropod lineages are needed before this concept can be accepted. The relatively easy accessibility of muscles and low effort procedure of immunofluorescence and AChE-staining should allow expanding this type of investigation to a broad range of arthropods and non-arthropod organisms. A phylogenetically intriguing feature appears to be also the loss of peripheral inhibition in holometabolous insects [19]. Whether this is indeed a synapomorphy of Holometabola could be verified by inspecting NMJs of more non-model insects. Broader taxon sampling could also clarify the possible value of general NMJ morphology as a phylogenetic character.

We have found first evidence for the presence of GABA in arthropod leg sensory neurons, expanding the potential neurotransmitter set in arthropod sensory systems. The challenging issue which sensory modalities are transduced by these neurons requires other experimental approaches including ultrastructural, electrophysiological, and behavioral methods.

## Abbreviations

ACh: Acetylcholine
AChE: Acetylcholinesterase
CNS: Central nervous system
EDTA: Ethylenediaminetetraacetic acid
GABA: Gamma-amino butytic acid
GAD: Glutamate decarboxylase
IR: Immunoreactivity
NMJ: Neuromuscular junction
PBS: Phosphate buffered saline

## References

[1] Otsuka M, Kravitz EA, Potter DD (1967). Physiological and chemical architecture of a lobster ganglion with particular reference to gamma-aminobutyrate and glutamate. J Neurophysiol.

[2] Usherwood PN, Machili P, Leaf G (1968). L-glutamate at insect excitatory nerve-muscle synapses. Nature.

[3] Jan LY, Jan YN (1976). L-glutamate as an excitatory transmitter at the Drosophila larval neuromuscular junction. J Physiol.

[4] Bicker G, Schäfer S, Ottersen OP, Storm-Mathisen J (1988). Glutamate-like immunoreactivity in identified neuronal populations of insect nervous systems. J Neurosci.

[5] Johansen J, Halpern ME, Johansen KM, Keshishian H (1989). Stereotypic morphology of glutamatergic synapses on identified muscle cells of Drosophila larvae. J Neurosci.

[6] Atwood HL, Klose MK, Squire LR (2009). Comparative biology of invertebrate neuromuscular junctions. Encyclopedia of neuroscience.

[7] Wallace BG, Muller KJ, Nicholls JG, Stent GS (1981). Neurotransmitter chemistry. Neurobiology of the leech. Cold Spring Harbor publications.

[8] Slater CR (2017). The structure of human neuromuscular junctions: some unanswered molecular questions. Int J Mol Sci.

[9] Martin RJ, Valkanov MA, Dale VM, Robertson AP, Murray I (1996). Electrophysiology of Ascaris muscle and anti-nematodal drug action. Parasitology.

[10] Nishimura K, Kitamura Y, Taniguchi T, Agata K (2010). Analysis of motor function modulated by cholinergic neurons in planarian Dugesia japonica. Neuroscience.

[11] Gerschenfeld HM (1973). Chemical transmission in invertebrate central nervous systems and neuromuscular junctions. Physiol Rev.

[12] Giribet G, Edgecombe GD (2012). Reevaluating the arthropod tree of life. Annu Rev Entomol.

[13] Stern M, Bicker G (2008). Mixed cholinergic/glutamatergic neuromuscular innervation of Onychophora: a combined histochemical/electrophysiological study. Cell Tissue Res.

[14] Usherwood PN, Grundfest H (1965). Peripheral inhibition in skeletal muscle of insects. Neurophysiology.

[15] Bräunig P, Schmäh M, Wolf H (2006). Common and specific inhibitory motor neurons innervate the intersegmental muscles in the locust thorax. J Exp Biol.

[16] Wolf H, Harzsch S (2002). Evolution of the arthropod neuromuscular system. 1. Arrangement of muscles and innervation in the walking legs of a scorpion: Vaejovis spinigerus (wood, 1863) (Vaejovidae, Scorpiones, Arachnida). Arthropod Struct Dev.

[17] Fabian-Fine R, Meisner S, Torkkeli PH, Meinertzhagen IA (2015). Co-localization of gamma-aminobutyric acid and glutamate in neurons of the spider central nervous system. Cell Tissue Res.

[18] Wolf H (2014). Inhibitory motoneurons in arthropod motor control: organisation, function, evolution. J Comp Physiol A.

[19] Witten JL, Truman JW (1998). Distribution of GABA-like immunoreactive neurons in insects suggests lineage homology. J Comp Neurol.

[20] Barker DL, Herbert E, Hildebrand JG, Kravitz EA (1972). Acetylcholine and lobster sensory neurons. J Physiol.

[21] Florey E (1973). Acetylcholine as sensory transmitter in Crustacea. New evidence from experiments demonstrating release. J Comp Physiol.

[22] Wang-Bennett LT, Sovan ML, Glantz RM (1988). Immunocytochemical studies of the distribution of acetylcholine in the crayfish brain. J Comp Neurol.

[23] Braun G, Mulloney B (1994). Acetylcholinesterase activity in neurons of crayfish abdominal ganglia. J Comp Neurol.

[24] Sanes JR, Hildebrand JG (1976). Acetylcholine and its metabolic enzymes in developing antennae of the moth, Manduca sexta. Dev Biol.

[25] Blagburn JM, Sattelle DB (1987). Nicotinic acetylcholine receptors on a cholinergic nerve terminal in the cockroach, Periplaneta americana. J Comp Physiol A.

[26] Leitch B, Pitman RM (1995). Modulation of transmitter release from the terminals of the locust wing stretch receptor neuron by muscarinic antagonists. J Neurobiol.

[27] Bicker G, Naujock M, Haase A (2004). Cellular expression patterns of acetylcholinesterase activity during grasshopper development. Cell Tissue Res.

[28] Clark J, Meisner S, Torkkeli PH (2005). Immunocytochemical localization of choline acetyltransferase and muscarinic ACh receptors in the antenna during development of the sphinx moth Manduca sexta. Cell Tissue Res.

[29] Gauglitz S, Pflüger HJ (2001). Cholinergic transmission via central synapses in the locust nervous system. J Comp Physiol A.

[30] Strausfeld NJ (2012). Arthropod brains. Evolution, functional elegance, and historical significance.

[31] Sombke A, Edgecombe GD (2014). Morphology and evolution of Myriapoda. Arthropod Struct Dev.

[32] Sombke A, Rosenberg J. Myriapoda. In: Schmidt-Rhaesa A, Harzsch S, Purschke G, editors. Structure and evolution of invertebrate nervous systems: Oxford University Press; 2016. p. 478–91.

[33] Von Holst E (1934). Über die 0rdnung und Unordnung der Beinbewegungen bei Hundertfüßern (Chilopoden). Pflügers Arch.

[34] Von Holst E (1943). Über relative Koordination bei Arthropoden (mit Vergleichsversuchen am Regenwurm). Pflügers Arch.

[35] Rilling G (1960). Zur Anatomie des braunen Steinläufers Lithobius forficatus L. (Chilopoda). Skelettmuskelsystem, peripheres Nervensystem und Sinnesorgane des Rumpfes. Zool Jb Anat.

[36] Sombke A, Stemme T. Serotonergic neurons in the ventral nerve cord of Chilopoda - a mandibulate pattern of individually identifiable neurons. Zoological Lett. 2017;3(9) 10.1186/s40851-017-0070-y. eCollection 2017. 10.1186/s40851-017-0070-y PMC5496589 28690866

[37] Karnovsky MJ, Roots LA (1964). “Direct-coloring” thiocholine method for cholinesterases. J Histochem Cytochem.

[38] Klagges BRE, Heimbeck G, Godenschwege TA, Hofbauer A, Pflugfelder GO, Reifegerste R, Reisch D, Schaupp M, Buchner S, Buchner E (1996). Invertebrate synapsins: a single gene codes for several isoforms in Drosophila. J Neurosci.

[39] Hoskins SG, Homberg U, Kingan TG, Christensen TA, Hildebrand JG (1986). Immunocytochemistry of GABA in the antennal lobes of the sphinx moth Manduca sexta. Cell Tissue Res.

[40] Sombke A, Harzsch S, Hansson BS. Organization of Deutocerebral Neuropils and Olfactory Behavior in the centipede Scutigera coleoptrata (Linnaeus, 1758) (Myriapoda: Chilopoda). Chem Senses 2011;36: 43–61. 10.1093/chemse/bjq096 20962283

[41] Stern M (2009). The PM1 neurons, movement sensitive centrifugal visual brain neurons in the locust: anatomy, physiology, and modulation by identified octopaminergic neurons. J Comp Physiol A.

[42] Battaglioli G, Liu H, Hauer CR, Martin DL (2005). Glutamate decarboxylase: loss of N-terminal segment does not affect homodimerization and determination of the oxidation state of cysteine residues. Neurochem Res.

[43] Heckmann R, Kutsch W (1995). Motor supply of the dorsal longitudinal muscles. II: comparison of motoneurone sets in Tracheata. Zoomorphology.

[44] Schmäh M, Wolf H (2003). Inhibitory motor neurones supply body wall muscles in the locust abdomen. J Exp Biol.

[45] Parnas I, Abbott BC, Shapiro B, Lang F (1968). Neuromuscular system of Limulus leg closer muscle. Comp Biochem Physiol.

[46] Rane SG, Wise GA (1987). Neuromuscular synaptic transmission in Limulus Polyphemus-I. Actions of aspartate, glutamate and the natural transmitter. Comp Biochem Physiol.

[47] Harzsch . S, Müller CHG,. Wolf H (2005). From variable to constant cell numbers: cellular characteristics of the arthropod nervous system argue against a sister-group relationship of Chelicerata and “Myriapoda” but favour the Mandibulata concept. Dev Genes Evol.

[48] Fabian-Fine R, Höger U, Seyfarth EA, Meinertzhagen IA (1999). Peripheral synapses at identified Mechanosensory neurons in spiders: three-dimensional reconstruction and GABA immunocytochemistry. J Neurosci.

[49] Watson AH, Storm-Mathisen J, Ottersen OP (1991). GABA and glutamate-like immunoreactivity in processes presynaptic to afferents from hair plates on the proximal joints of the locust leg. J Neurocytol.

[50] Kruangkum T, Chotwiwatthanakun C, Vanichviriyakit R, Tinikul Y, Anuracpreeda P, Wanichanon C, Hanna PJ, Sobhon P (2013). Structure of the olfactory receptor organs, their GABAergic neural pathways, and modulation of mating behavior, in the giant freshwater prawn, Macrobrachium rosenbergii. Microsc Res Tech.

[51] Csoknya M, Takács B, Koza A, Dénes V, Wilhelm M, Hiripi L, Kaslin J, Elekes K (2005). Neurochemical characterization of nervous elements innervating the body wall of earthworms (Lumbricus, Eisenia): immunohistochemical and pharmacological studies. Cell Tissue Res.

[52] Buchner E, Buchner S, Burg MG, Hofbauer A, Pak WL, Pollack I (1993). Histamine is a major mechanosensory neurotransmitter candidate in Drosophila melanogaster. Cell Tissue Res.

[53] Melzig J, Buchner S, Wiebel F, Wolf R, Burg M, Pak WL, Buchner E (1996). Genetic depletion of histamine from the nervous system of Drosophila eliminates specific visual and mechanosensory behavior. J Comp Physiol A.

[54] Fabian R, Seyfarth EA (1997). Acetylcholine and histamine are transmitter candidates in identifiable mechanosensitive neurons of the spider Cupiennius salei: an immunocytochemical study. Cell Tissue Res.

